# Utilization of titanium dioxide (TiO_2_) nanoparticles to improve functionality and mechanical performances of cotton fabric

**DOI:** 10.1016/j.heliyon.2024.e37899

**Published:** 2024-09-14

**Authors:** Imana Shahrin Tania, Mohammad Ali

**Affiliations:** a Department of Mechanical Engineering, Bangladesh University of Engineering and Technology, Dhaka-1000, Bangladesh; b Department of Wet Process Engineering, Bangladesh University of Textiles, Dhaka-1208, Bangladesh

**Keywords:** Cotton fabric, TiO_2_ nanoparticle, Wax-emulsifier, Mechanical properties, Antimicrobial, UV protection

## Abstract

This study aims to produce high-quality functional cotton fabric through the deposition of nano TiO_2_. Here nanoparticles are deposited by the pad-dry-cure method with different recipes formulated using an acrylic binder and wax emulsifier together with TiO_2_ nanoparticles to observe the optimal effect on the final quality. The treated fabric is characterized by SEM, EDS, XRD, and FTIR. To analyze the results, the antimicrobial properties, UV protection, and crease resistance are measured as functional properties. Furthermore, tensile and tearing strength, frictional resistance, bending length, abrasion resistance, and pilling are determined as mechanical properties. The results confirm that the binder significantly affects on nano deposition and improves the mentioned functional characteristics. However, the deposition of nano TiO_2_ has deteriorated cotton's mechanical properties, and that degradation is intensified by the use of binder. This degradation problem is overcome by using the emulsifier as an auxiliary since the recipe having an emulsifier improves all the mechanical qualities of nano-treated cotton fabric. Particularly, the sample containing a binder and an emulsifier (Nano TiO_2_-3) ensures up to 97 % UV-ray blockage, 93 % antibacterial activity, and 9 % increase in tensile strength.

## 1 Introduction

A nanoparticle is a state-of-the-art in advancement of science and technology that offers a wide range of unique possibilities, novel characteristics and enhances the typical function of the bulk substance [1]. The potentiality of nanoparticles lies in their enormous surface area rather than their volume, tremendous surface mobility, and a vast range of compositions, depending on the use or the product [2,3]. Among other nanoparticles, TiO_2_ has excellent prospects for use in textile finishing applications. TiO_2_ possesses some essential qualities that are advantageous to achieve the functional properties of cotton fabric. Because of its photocatalytic properties, as well as the fact that it is cost-effective, non-toxic, chemically stable, and generally recognized as safe, it is regarded as an alternative antibacterial agent [4].

In addition, TiO2 exhibits unique properties as a semiconducting transition metal with oxide material, allowing for its use in solar cells, antibacterial and antifungal agents, chemical sensors, electronics, and optics [5,6]. Due to its higher refractive index and semi-conductive nature, it can absorb UV rays and give higher protection against the UV Spectrum of solar light [7]. The textile sector takes advantage of this huge potential of nano TiO_2_ for advancing its product and the diversification of textiles to meet the demand of the modern market. TiO_2_ is mainly applied to the finishing section to impart desirable quality and to minimize the use of the extra chemical agent for various required attributes. The use of nanoparticles offers a minimal amount and multifunctional activity of treated fabric. On the other hand, globally cotton is widely used natural fiber due to its vast benefits, comfortability, biodegradability, easy dyeability, etc. Despite various good properties, there are some limitations of cotton fabric. As cotton fibrous material comes from a natural origin, it is susceptible to microorganism growth and damage with bacterial attack [8]. Besides, the hydrophilic nature and polysaccharide cellulosic structure of cotton fiber are responsible for the easy crease formation on cotton fabric [8,9]. In this instance, nano TiO_2_ may emerge as a groundbreaking nanoparticle to overcome those inherent drawbacks of cotton fabric as well as impart other functional properties like antimicrobial, UV protective, antistained, flame retardant, thermal conductive, etc. The nanoparticle has no sufficient attraction or fixation power towards textiles. So for the deposition and fixation of nanoparticles binder can be used. Metal oxide-based nanoparticles deposited with binder show a negative impact on the mechanical properties of cotton fabric [10]. Polyethylene is a softener and could make the fabric surface flexible, smooth, and soft. It has the evidence to improve the important mechanical properties of cotton fabric [11]. Our previous study found the usefulness of a binder on ZnO nanoparticle fixation on cotton fabric [10], where the binder caused the improvement of the functional property but reduced the mechanical properties, quantitively tensile strength was reduced by 10.4 % in warp and 12.23 % in the weft. However, the mechanical properties are also important from the service point of any fabric. In the current work, we have used the acrylic binder and a polyethylene wax emulsifier as the auxiliary for nano TiO_2_ deposition and compared the results with untreated fabric. The binder used herein is thermally cross-linkable, and aqueous-acrylate dispersion. It is usually used in pigment that facilitates the three-dimensional network with fiber as well as pigment during curing at high temperatures (150°C) [12]. Accordingly, for TiO_2_ nanoparticle fixation the binder (acrylic-based resin) performs a similar function, by polymerizing and fixing TiO_2_ nanoparticles into cotton fabric. Moreover, as the binder is self-cross-linkable it ensures a better hand feel of coated surface. Therefore, the effects of two essential auxiliaries (binder and wax emulsifier) on nano TiO_2_ treatment of cotton fabric are to be revealed and analyzed which are the key features of this study.

## 2 Experiments

### 2.1 Materials

Cotton woven fabric of a plain weave structure with a 20′S count and 168 GSM (gram/m^2^) is collected from the local export-oriented woven factory. The grey fabric is pretreated (desizing and bleaching) using the stranded recipe. Titanium tetrachloride (TiCl_4_ 99.9 % pure), and urea (CH_4_N_2_O, 99.8 % pure) are procured from Merck, Germany which is used for the synthesis of nanoparticles. Polyethylene wax emulsion named Jinlub Eco NP-825N (Character: nonionic-softener, water-soluble, yellowish liquid) is collected from Jintex, Taiwan. A thermally cross-linkable acrylic-based binder OB-45 (low viscous, white milk-type liquid) is provided by Fortune Top Pte Ltd., Taiwan.

### 2.2 Synthesis of TiO_2_

**Fig. 1:**
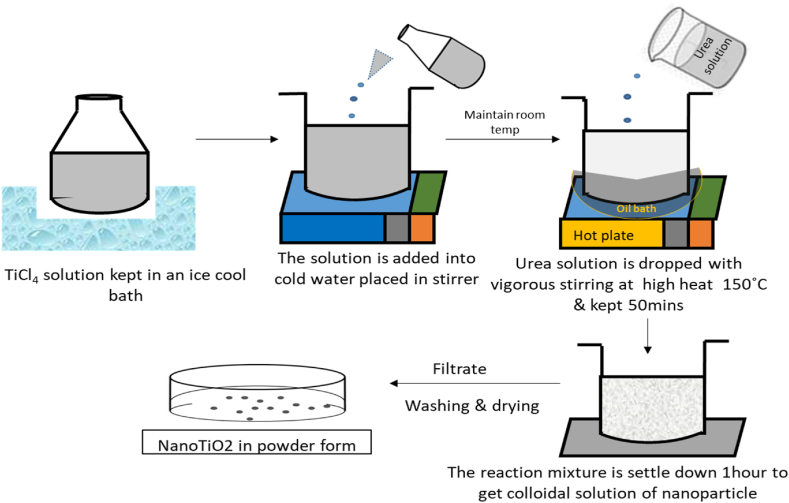
Experimental setup for nano TiO_2_ synthesis.

The nanoparticles are synthesized by slightly altering the method previously employed by the researchers [13,14]. The experimental setup for nano formation is shown in Fig. 1. For the synthesis, 100 ml of TiCl_4_ aqueous solution is kept in an ice-cool bath for 5 min. The solution is then added to 400 ml of cold distilled water, and the temperature is raised to 30° and kept for 20 min. In a separate biker, 52 g of urea is dissolved in 500 ml water and added to the TiCl_4_ solution in a dropwise manner. The reaction solution is raised to 150 °C and kept for 50 min. The reaction mixture is settled down for 1 h to get a colloidal solution of the nanoparticle. The colloidal solution is then filtrated, washed, and dried to obtain nanoparticles in powder form.

### 2.3 Coating of titanium dioxide nanoparticles on fabric

Cotton fabric is coated with TiO2 nanoparticles by using pad-dry and cure (mechanical thermo-fixation) method [17]. Three distinct recipes with the same amount of nanoparticles are used to coat the fabric surface. At first, the fabric is coated with 1 % TiO_2_ nanoparticle only, denoted as a sample of NanoTiO_2_-1. For the second coating, a 1 % TiO_2_ nanoparticle along with a 1 % binder (OB-45) is mixed by continuous stirring for 10 min, and then the fabric is coated with this solution, which is denoted as NanoTiO_2_-2. Finally, 1 % TiO_2_ nanoparticle, 10 g/L emulsifiers, and 1 % binder are mixed with the solution of propanol and stirred at 60° celsius temperature for 15 min. The fabric treated with the third solution is denoted as a sample of NanoTiO_2_-3. TiO_2_ nanoparticles are thermally fixed into the fabric during padding by maintaining a 73 % pick-up rate, drying at 90 °C for 10 min, and curing at 150 °C for 5 min.

### 2.4 Analysis and Measurements

#### 2.4.1 Evaluation of UV protection

Three types of UV radiation from solar light are available: i) 320–400 nm for UV-A, 280–320 nm for UV-B, and 200–280 nm for UV-C. Where UV-B and UV-C rays are the strongest and most potentially dangerous types of radiation, compared with UV-A. The UV-B rays are mostly responsible for wrinkles, aging, sunburn, and skin cancer. Fortunately, the ozone layer in the atmosphere absorbs the majority of the UV-C rays, which are the most damaging and the shortest. Thus, UV-A and UV-B regions are the evaluating areas for sun protection [18]. Using functional textile materials as protection against UV rays is one of the most significant strategies to avoid skin cancer and other related skin problems caused by UV light from the sun [19]. The fabric ensures protection by absorbing and blocking the penetration of UV rays [20]. In this research, the PerkinElmer UV visible machine, made in the USA, is used to assess the UV protection of both treated and untreated fabric. The machine provides a transmittance curve of every sample, and by evaluating the transmittance curve, the UV protection properties is found.

#### 2.4.2 Evaluation of antimicrobial activity

To determine the antimicrobial activity a standard test method is followed [21]. By using the colony counting method, the investigation is conducted against a gram-positive named *S. Aureus* and a gram-negative bacteria called *E. coli*. The results of the test are stated as the bacterial reduction percentage (R%). This method is designed for surfaces with a 50–100 % reduction capability during the necessary contact period [22]. The formula shown in Equation (2) is used to determine the percent of bacteria killed within the selected time.

(2) Thebacterialreductionpercentage(R%)=No−NtNo×100

No is the number of colonies in control, and Nt is the number of colonies after the selected hour of introducing samples. Here the sample is examined for 1 h and 24 h of contact time. The treated sample is submerged in a washing solution containing non-ionic detergent (2 g/L) to assess the durability of nano-TiO_2_-treated cotton fabric against repeated laundering [23]. At 50 °C, the samples are agitated for 15 min. After that, it is rinsed and dried. This method is performed five and ten times, and the washed sample's antibacterial property is then determined.

#### 2.4.3 Crease resistance

A crease is a broken line, mark, or fold on fabric that results from precise folding. It is found on the fabric surface when an area of the fabric is stretched past its elastic recovery due to distortions for different purposes [24]. On the other hand, crease resistance is a characteristic of fabric that resists fabric from creasing or deforming. **Crease resistance is** normally measured by the recovery angle (degree) of the fabric. The more angle of recovery specifies more crease resistance of fabric. It is also regarded as a quantitative amount of the easy-care property of a fabric. In our present research, the crease recovery angle of untreated and nano-TiO_2_-treated fabric is dignified using an American standard test method [25].

#### 2.4.4 Mechanical properties

Two important mechanical properties of the treated and untreated fabric are determined by following the standard text method. Tensile strength is measured as per the ISO 13934-1:2013 test method, and tearing strength is measured as per ISO13937-2:2000. The test is conducted using Jeams Heals' Titan universal strength tester. During fabric strength measurements the dimensions are taken in both the warp and weft directions. Three samples are measured, and the average values are calculated. ASTM D1388 uses a Shirley stiffness tester to assess fabric stiffness expressed in bending length. The frictional property is also assessed using a Fabric touch tester from SDL Atlas in the United States. Before every test, the experimental samples are conditioned in a conditioning laboratory maintaining a standard atmosphere (Temp: 20 °C, Relative humidity: 65.2 %, Times: 12 h).

#### 2.4.5 Characterization of Treated Fabric

SEM, EDS XRD, and FRIT are used to characterize the treated fabric. The instrumental specification of SEM and XRD analysis has been mentioned in the previous section 2.3. The structural changes of cotton fabric and the bonding of cellulose fiber with nano TiO_2_ are monitored using Fourier Transform Infrared Spectroscopy (FTIR). Untreated and nano TiO_2_-treated fabrics are characterized using an ATR/FTIR machine from Frontier, PerkinElmer, USA. In this machine, the scanning range for testing is selected as 4000-1000 cm^−1^ [26]. Igor Pro software is used to compute XRD and FTIR data.

## 3 Results and discussion

### 3.1 Characterizations of nanoparticle

**Fig. 2:**
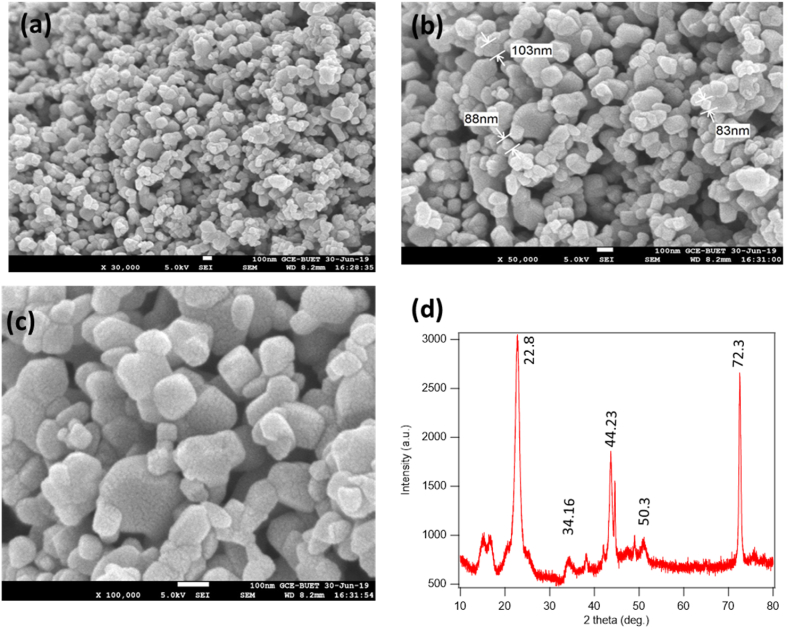
SEM images of TiO_2_ nanoparticles in different magnifications a) × 30,000, (b) × 50,000, and (c) × 100,000; **(d)** XRD pattern of synthesized TiO_2_.

High-resolution SEM (Scanning Electron Microscopy) and XRD (X-ray diffraction) studies are done to characterize the synthesized nanoparticles. These analyses reveal the approximate size, surface characteristics, and other features of discrete nanoparticles. SEM analysis is done by JSM-6700F, from Tokyo, Japan. Prior to SEM examination, the sample is prepared for testing by auto fine coater (Jeol JFC-1600 Japan). With this machine, a 75 nm thin platinum layer is created on our non-conductive cotton fabric. The obtained image found from SEM analysis is shown in Fig. 2(a and b,c) which displays individual nanoparticles in magnifications of 30,000, 50,000, and 10,0000. The size measuring software detects the estimated size of the nanoparticle and is shown in Fig. 2 (b). During SEM analysis, a sample is subjected to a tightly focused scanning electron beam which producing a huge number of secondary electrons whose power is controlled by surface topography [1]. As a consequence, the particles of various shapes and sizes are visible in the SEM images.

The X-ray diffractometer obtains the crystalline shape and size: Phillips, X'pert PRO, Holland. The XRD pattern of TiO_2_ nanoparticles is shown in Fig. 2(d). The scanning rate is 8°/min in the 2θ range of 20–80°, where, θ is the angle between the incident beam and the crystallographic reflecting plane, and 2θ is the angle between the transmitted beam and reflected beam. The size of the nanoparticles is determined by using the following Deby-Scherrer formula as in Equation (1)

(1) ParticleSize=(0.9xλ)/(dcosθ)

where, λ = 1.54060 Å, 0.9 × λ = 1.38654, θ = 2θ/2, d is the full width at the half maximum intensity of the peak. From this Equation, it is found that the calculated average size of nano TiO_2_ is 80 nm. The XRD pattern obtained in Fig. 2 (d) is [2θ = 22.8°, 34.16°, 44.23°,50.3°, 72.3°] consistent with the JCPDS card no. 21-1272 (anatase TiO_2_) and the XRD pattern of TiO_2_ nanoparticles have been reported previously [15,16]. The peak intensity of the XRD image indicates that the synthesized nanoparticles are crystalline.

### 3.2 UV protection

**Fig. 3:**
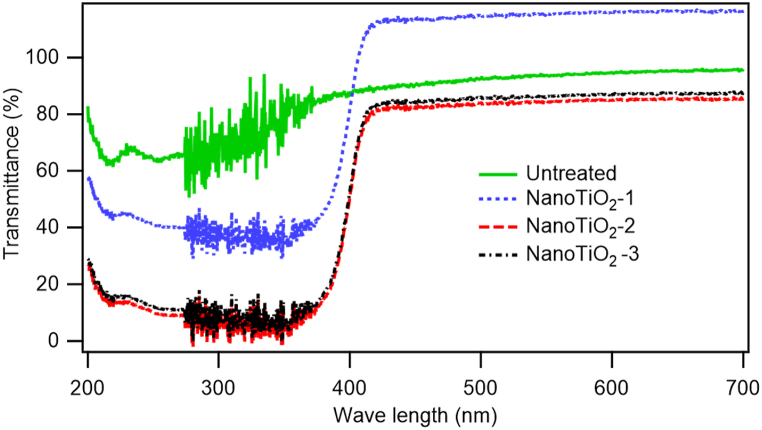
UV transmittance curve of untreated and nano TiO2-coated cotton fabrics.

The UV protection property of a fabric is expressed by the transmittance % of incident solar light of 200–700 nm. The high transmittance indicates low UV blocking and the low transmittance indicates higher UV protection. Fig. 3 represents the UV transmittance curve and Table 1 represents quantitative Transmittance % against three different wavelengths of incidental solar light. According to Fig. 3, each sample having TiO_2_ shows a certain amount of VV protection. Due to the use of binder on samples NanoTiO_2_-2 and NanoTiO_2_-3 give lower transmittance i.e., the higher UV blockage. It can be found that around 92–96 % of UV rays are blocked on the UV-A and UV-B regions by the treated samples, NanoTiO_2_-2 and NanoTiO_2_-3. Based on the literature [27] and Australian/New Zealand standards AS/NZS 4399:2017, the usual UPF ranges are also estimated and reported in Table 1, which shows good UV protection of nano TiO2-coated fabric with binder and emulsifier. The UV protection ability of TiO_2_ nanoparticles has been demonstrated by the earlier work of other researchers. For instance, Xin et al. [28] looked into the impacts of TiO_2_ on the UV-blocking qualities of cotton fabric and found positive results. Yu et al. [29] used around 50 nm TiO_2_ nanoparticle as a UV agent on cotton fabric in combination with polyvinyl pyrrolidone (PVP) and obtained a UPF value of 15–35, which is similar to our findings. Chen et al. [30] also found excellent UV-blocking properties of nano-TiO_2_-deposited cotton fabric. Additionally, it has been noticed that the fabric treated with TiO_2_ nano-deposition demonstrates enhanced UV protection capabilities when compared to the fabric treated with nano ZnO deposition, as supported by a comparative analysis conducted in one of our prior studies [31]. Therefore, it is anticipated that the application of TiO_2_ nanoparticles is preferred for the nanocoating of fabrics that demand sun protection.

**Table 1:** UV transmittance (%) of untreated and treated fabric in different wavelengths with UPF (ultraviolet protection factor) values.

Sample	Transmittance (%)			Measured UPF concerning Average Transmittance%	UV protection category^a^
	UV-A (320-400) nm	UV-B (280-320) nm	UV-C (200-280) nm		
Untreated	70 ± 3	60 ± 2	52 ± 2	5 ± 2	Poor
NanoTiO_2_-1	35 ± 2	37 ± 2	41 ± 4	10 ± 3	Moderate
Nano TiO_2_-2	6 ± 2	3 ± 1	8 ± 4	35 ± 4	Good
Nano TiO_2_-3	7 ± 3	3 ± 1	9 ± 3	33 ± 3	Good

a Category of UV protection assessed as per AS/NZS 4399:2017 [32].

### 3.3 Antimicrobial activity

**Fig. 4:**
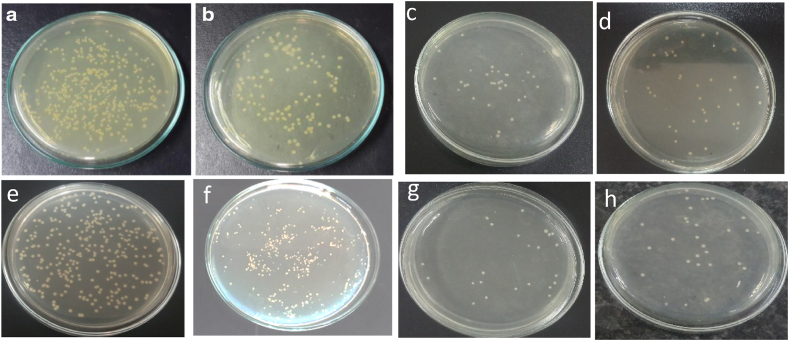
Bacterial reduction disk images of untreated and nano-treated fabric after 24 h against *S. aureus,* [ a) Untreated fabric b) NanoTiO_2_-1 c) NanoTiO_2_-2, and d) NanoTiO_2_-3] and against *E.Coli* [e) Untreated fabric f) NanoTiO_2_-1, g) NanoTiO_2_-2, and h) NanoTiO_2_-3.

The antimicrobial activity is expressed as a percentage of bacterial reduction in Table 2, and the disk image of bacterial reduction is shown in Fig. 4. The images show the clear difference in bacterial growth of untreated and nano TiO_2_ deposited fabric. Fig. 4 (a, e) represents more bacterial growth responsible for untreated fabric against *S.aureus* and *E.coli,* respectively. The others in Fig. 4(b and c,d) show the decrease of bacterial growth compared with untreated fabric responsible for nano TiO_2_ deposited fabric against *S.aureus.* The disk image for the action of *E.coli* also shows a similar trend of decreasing bacterial growth for treated fabric as in Fig. 4(f and g,h). The binder deposited more TiO_2_ and consequently, sample NanoTiO_2_-2 gives more bacterial reduction that can be found in both Fig. 4 and Table 2. Quantitively, TiO_2_ is a good antimicrobial agent, and binder has a significant effect on nano deposition [10] and the antimicrobial activity of treated fabric. The wash durability of antimicrobial activity is also checked and shows appreciable results. NanoTiO_2_-2 is capable of showing admirable antibacterial action for 24 h, as evidenced by a 94 % reduction. The durability of antibacterial activity is also found good. The sample NanoTiO_2_-3 shows similar results of antimicrobial activity and wash durability. According to a literature review, TiO_2_ deposition has a significant antibacterial effect on textile fabric [33]. The antimicrobial characteristics of TiO_2_ are attributed to the generation of reactive oxygen species (ROS), including hydroxyl radicals (OH) and superoxide radicals (O_2_), which exhibit antimicrobial action [34,35]. Our finding of antibacterial activity is consistent with that of other earlier researchers [36].

**Table 2:** Bacterial reduction of untreated and nano TiO_2_ treated fabric against *S. aureus* and *E. coli* after 1 h and 24 h contact time.

Sample	Bacterial reduction R% after 1-h contact time		Bacterial reduction R% after 24-h contact time	
	*S. aureus*	*E. coli*	*S. aureus*	*E. coli*
**Untreated**	–	–	–	–
**NanoTiO**_**2**_**-1**				
Unwashed	35.43 ± 0.0	43.14 ± 0.0	51.20 ± 0.0	50.11 ± 0.5
5 Wash	28.12 ± 1.0	23.11 ± 0.0	37.40 ± 0.5	36.10 ± 0.0
10 Wash	16.41 ± 1.0	19.23 ± 1.0	34.14 ± 1.0	20.6 ± 0.5
**NanoTiO**_**2**_**-2**				
Unwashed	78.23 ± 0.0	75.22 ± 0.0	93.50 ± 0.0	86.15 ± 0.0
5 wash	76.20 ± 0.5	74.50 ± 1.0	87.16 ± 0.0	84.13 ± 0.5
10 wash	74.16 ± 0.5	73.00 ± 0.5	86.01 ± 1.0	84.1 ± 0.0
**NanoTiO**_**2**_**-3**				
Unwashed	76.23 ± 0.5	74.22 ± 0.0	90.50 ± 0.0	85.15 ± 1.0
5 wash	74.20 ± 0.0	73.50 ± 0.5	85.16 ± 1.0	84.13 ± 1.0
10 wash	73.56 ± 0.0	72.00 ± 0.0	83.41 ± 1.0	83.01 ± 0.5

### 3.4 Crease resistance

**Fig. 5:**
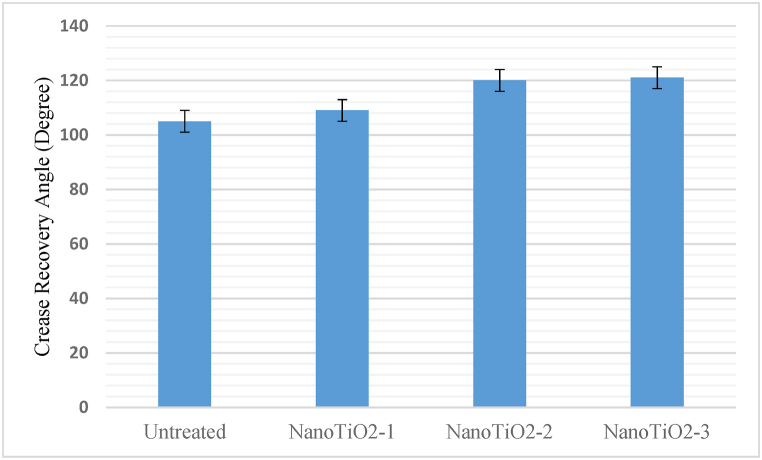
Crease recovery power of untreated and nano TiO_2_ deposited fabric.

Crease resistance is expressed by the crease recovery angle of untreated and treated fabric measured by the American standard test method [20]. It also represents the ease care property of a fabric. More recovery angle means more resistance to creases. The crease recovery angle of several samples is depicted in Fig. 5 [Sample size for the measurement is 10 and the standard error is ±1]. The graph shows that TiO_2_ is an effective crease-resistance agent. As a result, every sample with TiO_2_ particles has a higher crease resistance than the untreated cloth sample. For example, NanoTiO_2_-1 displays a 109-degree recovery angle, NanoTiO_2_-2 displays 120 and NanoTiO_2_-3 displays the greatest which is a 121-degree recovery angle. Therefore, the crease recovery angle of TiO_2_ nano treatment becomes higher by binder and polyethylene emulsifier. Binder application increases wrinkle resistance by 14 % compared to untreated cloth. The emulsifier also increases the resistance of TiO_2_ nano-treated fabric by 15.23 %. However, TiO_2_ has been used by other researchers as a crease-resistance-improving agent. For example, Yuen et al. [37] used nano titanium dioxide as a co-catalyst compound for their non-formaldehyde wrinkle-resistant treatment on cotton fabric. The application of TiO_2_ nanoparticles improved the crease recovery angle, according to their findings.

### 3.5 Mechanical Property Analysis

For the analysis of mechanical properties, the tensile strength, tearing strength, elongation, abrasion resistance, bending length, frictional resistance, and pilling are considered and calculated. The tensile strength shown in Table 3 looks a little decreasing than that of untreated fabric. The sample NanoTiO_2_-1 gives 6.8 %, NanoTiO_2_-2 gives 10.8 % reduction of tensile strength. However the sample NanoTiO_2_- 3 shows 801.11N of strength, around 9 % more than that of untreated fabric. This sample contains NanoTiO_2_ with a binder and emulsifier. So, emulsifier has a significant effect on the improvement of fabric tensile strength. At the same time, the binder has a negative effect on the strength. Fabric elongation and tearing follow a similar trend of decreased strength for samples NanoTiO_2_-1 and NanoTiO_2_-2. Again, the sample NanoTiO_2_-3 shows an improvement of 5.6 % warp elongation and 3.4 % weft elongation. It improves 10 % of tearing strength in the warp and 7.5 % in the weft direction.

**Table 3:** Mechanical properties of untreated and nano TiO_2_-treated cotton fabric.

Sample	Tensile strength Breaking force(N)		Elongation (%)		Tearing strength braking force (N)		Bending length (cm)	Frictional coefficient	
	Warp	Weft	Warp	Weft	Warp	Weft		Static	Kinetic
Untreated	730.52 ± 9	572.60 ± 6	21.10 ± 3	14.75 ± 3	9.11 ± 2	4.3 ± 2	1.8 ± 1	0.35	0.32
NanoTiO_2_-1	680.12 ± 6	546.32 ± 5	19.1 ± 3	13.11 ± 3	7.8 ± 3	3.51 ± 1	1.7 ± 1	0.36	0.31
NanoTiO_2_-2	651.22 ± 7	498.13 ± 5	18.2 ± 3	13.0 ± 3	5.6 ± 2	3.0 ± 2	1.7 ± 1	0.36	0.31
NanoTiO_2_-3	801.11 ± 7	611.24 ± 6	22.3 ± 4	15.26 ± 4	10.1 ± 3	7.56 ± 3	1.4 ± 1	0.30	0.28

Bending length is the measure of the stiffness of fabric expressed as the length in cm as can be found in Table 3. The results show that the treated fabric decreases in bending length than that of untreated fabric. The use of binder and emulsifier also shows a lower amount of bending length than that of untreated fabric. So it indicates that the use of emulsifier and nano TiO_2_ make the fabric less stiff by imparting flexibility and smoothness. This result is consistent with the findings of other studies. Yuen et al. [37] observed that utilizing nano TiO_2_ reduced tensile and tearing strength while decreasing bending length, yielding fabric smoothness.

The frictional resistance is reduced due to nano TiO_2_ deposition as the emulsifier imparts smoothness on the fabric and shows further reduction of both static and kinetic friction of treated fabric. As a result, it provides a high-quality treated fabric that facilitates fabric processing during cutting and sewing, resulting in a higher production rate.

TiO_2_ nanoparticles are distinctive by nature [38], and their coating ensures a range of capabilities that demonstrate promising potential [39]. Consequently adding nano-TiO_2_ to cotton fabric enhances their functional characteristics like UV protection, antibacterial activity, and crease resistance. However, as the nanoparticles are immobilized on the cotton by pad-dry and cure (at a high temperature), the fabric's surface becomes modified, the intermolecular gap of the fibers reduces and, as a result, the mechanical characteristics are adversely impacted. A recent paper supports the assertion that cotton fabric's mechanical properties are decreased by metal oxide nanoparticle inclusion [40].

### 3.6 Surface characterization of nano-deposited fabric

**Fig. 6:**
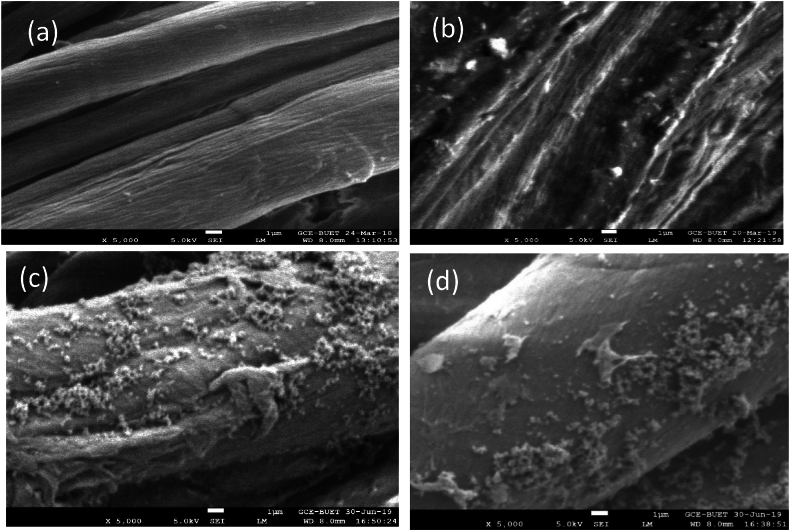
SEM image of a) untreated, b) NanoTiO_2_-1 c) NanoTiO_2_-2 d) NanoTiO_2_-3.

**Fig. 7:**
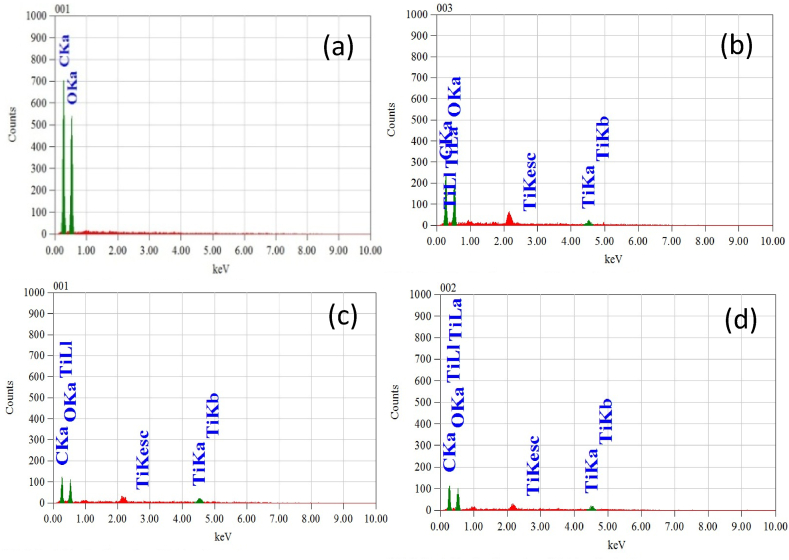
EDS image of a) Untreated, b) NanoTiO_2_-1 c) NanoTiO_2_-2 d) NanoTiO_2_-3.

The surface morphology of untreated and treated fabric is analyzed by SEM image and shown in Fig. 6(a–d). It can be found that the untreated fabric is free of nano deposition, whereas every treated fabric sample NanoTiO2-1, NanoTiO_2_-2, and NanoTiO_2_-3 has significant nanoparticle deposition. The sample NanoTiO_2_-2, where the binder (brand name: OB-45) is used for fixation, shows the highest amount of nanoparticle deposition. The third sample, NanoTiO_2_-3, having an emulsifier and binder with nano TiO_2,_ also indicates a higher amount of nano deposition. It confirms the nano deposition on treated fabric which increases for using binder (OB-45) as nano fixing agents. This is supported by the previous study that confirms that the binder improves bonding and imparts time longevity of nano deposition [40]. The elemental composition of the treated and untreated fabric is determined by EDS (Energy Dispersive Spectroscopy), and obtained images are placed in Fig. 7. The mass composition of a particular point in percentages is given in Table 4. It is observed that the untreated fabric shows the peaks corresponding to the carbon and oxygen that are the main elements of cellulose structure in Fig. 7(a). The treated fabric shows the corresponding peaks of titanium carbon, and oxygen in Fig. 7(b and c,d).

**Table 4:** Elemental composition of untreated and nano-treated samples obtained from EDX.

Elemental mass of different samples			
Sample	Mass (%)		
	Carbon	Oxygen	Titanium
Untreated	51.37	48.63	00
NanoTiO_2_-1	70.12	24.79	9.24
NanoTiO_2_-2	60.87	15.85	17.92
NanoTiO_2_-3	63.81	19.63	15.29

### 3.7 XRD analysis

**Fig. 8:**
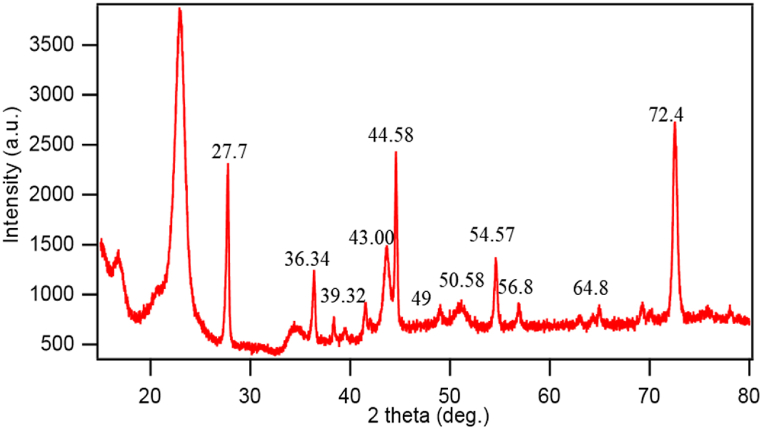
XRD pattern of nano TiO_2_ treated fabric sample NanoTiO_2_-3.

The XRD pattern of nano-treated fabric for sample NanoTiO_2_-3 is shown in Fig. 8. The figure shows the sharp peaks at 2θ = 27.7, 36.34, 38.35, 39.32, 41.49, 43.0, 44.58, 49, 50.8, 54.57, 56.8, 56.8, 64.86, 78° that is the corresponding peak of TiO_2_ nanoparticles. Similar peaks of TiO_2_ nanoparticles were found in the literature [41,42]. The sharp peaks in the figure signify the crystalline shape of the deposited nanoparticle. Besides, the size of the nanoparticle is determined by using Bragg's Equation as mentioned in section 2.3 (Equation (1)), which found that the particle size is 80 nm by analyzing the peak of the curve.

### 3.8 FTIR analysis

**Fig. 9:**
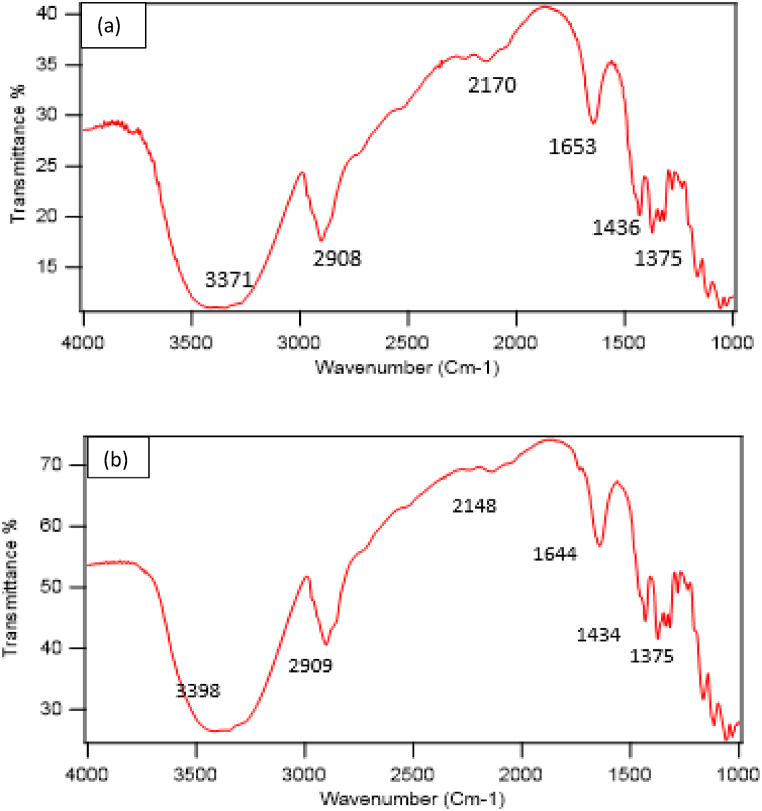
FTIR spectrum of a) untreated and b) nano TiO_2_-treated fabric.

The chemical bonding with a particular group of untreated and nano-treated fabric is revealed by FTIR analysis. The FTIR spectrum is shown in Fig. 9(a and b). The obtained peaks for untreated fabric are analyzed and found the band at 3371 cm^−1^ which is due to the O–H vibration 2908 cm^−1^ for C-H stretching, 2170 cm^−1^, 1653 cm^−1^, 1436 cm^−1^,1375 cm^−1^ for C-H, O-H, C-O, and C=O stretching which is the prominent characteristic peaks of a cellulose structure of cotton fabric [43]. Almost similar peaks are also found for the treated fabric shown in Fig. 9 (b). Compared to the untreated fabric, only a very slight deviation is located in the peaks; however, the first peak is found in the ranges 3100–3700 cm^−1^, which is accountable for the O-H vibration of cellulose. Each of the other peaks is located within the range of stretching for C-H, C-O, C-H, and C-O-C, which is also the characteristic bond of cotton fabric [7]. Thus, the treatment of TiO_2_ nanoparticles on cotton does not alter the characteristics bond of cotton fabric as it is regarded as a coating and cannot hamper the cellulose structure.

## 4 Conclusions

The synthesized TiO_2_ is applied to cotton fabric along with two auxiliaries named acrylic binder and wax emulsifier. The surface of the nano-deposited fabric is characterized by SEM, and elemental mass is determined by EDS. This analysis confirms the immobilization of TiO_2_ nanoparticles on cotton fabric. It is found that the binder has significantly increased the amount of nano deposition. The three functional charecteristics namely UV protection, antimicrobial activity, and crease resistance are evaluated, and found that each property is influenced by the use of a binder and nano-TiO_2_. The investigation reveals that NanoTiO_2_-2 and NanoTiO_2_-3 have outstanding antibacterial activities, adequate UV protection, and good crease resistance. It can be mentioned that the binder induces more nano deposition on the surface of cotton fabric, and consequently, the functional characteristics of these two samples have been improved.

In contrast, nano TiO_2_ causes the degradation of mechanical properties in samples NanoTiO_2_-1 and NanoTiO_2_-2, where no emulsifier is used. On the other hand, for the sample NanoTiO_2_-3, the emulsifier improves all the mechanical performance. At the same time, the emulsifier does not influence the functional attributes of the sample, as observed by the outcomes of NanoTiO_2_-3. Finally, it has been demonstrated that nano TiO_2_ should be utilized in conjunction with a binder and an emulsifier to provide the best functional and mechanical performance.

## Data availability statement

Few raw Data of XRD and FTIR are available which can be provided on request.

## CRediT authorship contribution statement

**Imana Shahrin Tania:** Writing – original draft, Validation, Methodology, Investigation, Formal analysis, Data curation, Conceptualization. **Mohammad Ali:** Writing – review & editing, Supervision, Project administration, Investigation.

## Declaration of competing interest

The authors declare that they have no known competing financial interests or personal relationships that could have appeared to influence the work reported in this paper.

## Abbreviations

SEM: Scanning Electron Microscopy
XRD: X-Ray Dispersive Spectroscopy
EDS: Energy Dispersive Spectroscopy
FTIR: Fourier Transform Infrared Spectroscopy
UV: ray - Ultraviolet radiation
R%: Reduction percentage of bacteria
AATCC: The American Association of Textile Chemists and Colorists
